# A lay-counsellor delivered brief psychological treatment for men with comorbid Alcohol Use Disorder and depression in primary care: Secondary analysis of data from a randomized controlled trial

**DOI:** 10.1016/j.drugalcdep.2021.108961

**Published:** 2021-10-01

**Authors:** Jasper Synowski, Helen A. Weiss, Richard Velleman, Vikram Patel, Abhijit Nadkarni

**Affiliations:** a Sangath, House no. 451 (168), Socorro Village, Bardez-Goa, Goa, 403501, India; b Charité – Universitätsmedizin, Charitéplatz 1, Berlin, 10117, Germany; c London School of Hygiene & Tropical Medicine, Keppel Street, London, WC1E 7HT, UK; d Department of Psychology, University of Bath, Claverton Down, Bath, BA2 7AY, UK; e Department of Global Health and Social Medicine, Harvard Medical School, Boston, MA, USA

**Keywords:** Comorbidity, Alcohol Use Disorder, Depression, Brief intervention, Task sharing

## Abstract

**Background:**

We investigated the feasibility, acceptability, safety, and preliminary effectiveness of the Counselling for Alcohol Problems (CAP) psychological intervention delivered by non-specialist health workers (NSHW) to participants with alcohol use disorder (AUD) and comorbid depression in primary care.

**Methods:**

We used data from a single blind randomised controlled trial conducted in ten primary health care centres in Goa, India. Adult male harmful or dependent drinkers with or without depression were randomized (1:1) to receive either CAP & enhanced usual care (EUC) or EUC only. Process indicators such as the number of completed counselling sessions were assessed and compared between comorbid and non-comorbid participants. Remission from AUD and depression along with abstinence were measured at 3 and 12 months post randomisation. Analyses were on an intention-to-treat basis, employing multivariable regression analyses.

**Results:**

271 participants had symptoms of comorbid depression; 241 did not. Both groups completed a similar number of counselling sessions (adjusted Mean Difference 0.05, 95 %CI −0.24–0.34;p = 0.72). Among comorbid participants, CAP did not lead to more frequent adverse events compared to EUC only (adjusted Odds Ratio [aOR] 0.84, 0.43–1.64;p = 0.62), and there was no evidence for an effect of CAP on remission from AUD or depression at 3 months (aOR 1.51, 0.84–2.74;p = 0.17 and aOR 0.74, 0.43–1.27;p = 0.28) and 12 months follow-up, respectively (aOR 1.69, 0.96–3.01;p = 0.08 and aOR 1.08, 0.62–1.87;p = 0.79).

**Conclusions:**

Brief therapies like CAP can be safely delivered by NSHWs to patients with comorbid AUD and depression, but their effectiveness may be limited and requires further investigation.

## Highlights

• Brief therapy can be safely delivered to patients with AUD and comorbid depression.
• Treatment acceptability and engagement comparable to patients with AUD only.
• No indication of effect among comorbid patients; larger studies needed.

## 1 Introduction

Alcohol Use Disorder (AUD) and depression account for a substantial burden of disease worldwide (Patel et al., 2016), and have a significant impact on a person’s quality of life, resulting in a reduced ability to participate in social as well as occupational life. In India, depression and AUD are both relatively common in men: in the 2016 National Mental Health Survey (NMHS), the prevalence of depression and AUD was estimated to be 2.4 % and 9.1 % respectively (Gururaj et al., 2016). The experience of these disorders appears to be linked - comorbid depression and AUD was recently found to be the most common dual mental comorbidity in India (Gururaj et al., 2016), and there is evidence from high-income settings suggesting that the presence of either AUD or depression significantly increases the risk of the other disorder (Boden and Fergusson, 2011; Crum et al., 2008; Fergusson et al., 2009). The treatment needs for this group are especially high because the negative impact of these comorbid disorders greatly exceeds the effect of a single disorder (Carton et al., 2018; Saatcioglu et al., 2008). This comorbidity results in substantial costs to society due to high health-care utilisation, sub-optimal treatment outcomes, and lost productivity (Chisholm et al., 2003; Rehm et al., 2009).

There is accumulating evidence that integrated psychological interventions can effectively treat both AUD and depression for people with comorbidity (Baker et al., 2012). However, some psychopathological symptoms comorbid with AUD are potentially due to the direct psychotropic effect of alcohol, and comorbid depressive symptoms significantly subside following a 4 to 5-week period of abstinence (Liappas et al., 2002). Hence, it is plausible that delivering evidence-based interventions only targeting AUD to patients with comorbid depression may have a beneficial effect in accelerating improvement of depression symptoms. The existing evidence for this is mixed, with psychological interventions targeting alcohol use in comorbid AUD and depression leading to improved drinking outcomes but no effect on the symptoms of depression in some studies (Satre et al., 2016) and not having any effect even on the drinking outcomes in others (Grothues et al., 2008). On this background of inconclusive and conflicting evidence it is difficult to make a definitive recommendation. Thus, guidelines such as those by the International Society of Addictions Medicine, and others suggest treating the substance use disorder first and to initiate treatment for depression only if it persists after a period of abstinence or reduced drinking (Hassan, 2017; El-Guebaly et al., 2020).

Access to evidence-based care remains limited due to systemic barriers such as availability, affordability, provider skills and knowledge (Saunders et al., 2006). Hence the treatment gap for AUD and depression in India is estimated to be more than 85 % for each disorder (Gururaj et al., 2016). One way to overcome health system barriers, especially in resource-poor settings, is to deliver interventions through task sharing (i.e. rational redistribution of tasks among health workforce teams) using non-specialist health workers (NSHW) to address the shortage of specialist human resources.

There is growing evidence supporting the effectiveness of NSHW-delivered interventions for AUDs as well as depression in low- and middle-incomce countries (LMICs) (van Ginneken et al., 2013). Furthermore, there is modest evidence supporting counselling for AUD in primary care settings (Jonas et al., 2012). There is clear evidence which shows that specific, well defined psychosocial therapies such as brief counselling, motivational enhancement therapy, community reinforcement approach, guided self-change, behaviour contracting, and social skills training are effective interventions for AUDs, together with some pharmacological interventions (Carvalho et al., 2019).

Thus, although there is good evidence for psychosocial treatments for AUD and modest and emerging evidence of effective NSHW delivered interventions for AUD in primary care, there is a lack of evidence on whether such interventions for AUD delivered by NSHWs would be effective in patients with comorbid AUD and depression; and to the best of our knowledge, this has not been tested in low-resource settings. The aim of this paper is to address this using data from the PREMIUM (Program for Effective Mental Health Interventions in Under-Resourced Health Systems) randomized controlled trial (RCT) conducted in Goa, India (Patel et al., 2014), in which the Counselling for Alcohol Problems (CAP) intervention was tested in harmful and dependent drinkers (Nadkarni et al., 2019, 2017, 2015). In this paper, we analyse data from the CAP trial to investigate a) the feasibility of identifying and recruiting men with probable AUD and comorbid depression in primary care, b) the feasibility of delivering a brief treatment for such patients by lay counsellors in primary care, c) the acceptability and safety of the treatment, and d) preliminary evidence of impact of the treatment on drinking and depression. We compare the data of comorbid participants in the CAP trial with the data of those with AUD without comorbid depression to explore whether the effect of the CAP intervention is modified by the presence of comorbidity.

## 2 Methods

### 2.1 Registration and ethical approval

A detailed description of the PREMIUM trial can be found in the study protocol (ISRCTN76465238) and in previous publications (Nadkarni et al., 2017; Patel et al., 2017, 2014). The study was approved by the Institutional Review Boards of the London School of Hygiene and Tropical Medicine, the Indian Council of Medical Research and Sangath.

### 2.2 Study setting

The trial was conducted in Goa, a small state in Western India. Goa is among the more developed states of India, and alcohol is available at comparatively cheap rates. The prevalence of AUD and common mental disorders in primary care in Goa has been reported as 8.2 % and 18.8 % respectively (D’Costa et al., 2006; Patel et al., 1998).

### 2.3 Study design and participants

The PREMIUM trial was implemented as a parallel-arm, single blind individually randomised controlled trial in 10 primary health centres (PHCs) across Goa. It was originally designed to test the effectiveness of two brief treatments – one (CAP) for patients with AUD, and a second one (the Healthy Activity Program (HAP)) for patients with depression – in two different trials (referred to as the CAP trial (Nadkarni et al., 2017) and the HAP trial (Patel et al., 2017) in the following). For the current study, only data of patients in the CAP trial were analysed.

For the CAP trial, patients at PHCs were eligible for screening if they identified as male, since the prevalence of AUD in women is very low in India (Gururaj et al., 2016). Additionally, patients needed to be 18–65 years old, reside in the respective PHC’s catchment area and plan to do so for the following year, be able to to communicate clearly, and understand one of the four languages the intervention was offered in. Patients presenting with medical emergencies were excluded. Screening for AUD was conducted using the AUDIT (Alcohol Use Disorders Identification Test) screening tool, a WHO-developed screening questionnaire which comprises of 10 items and has been validated for use in India (Pal et al., 2004). For the outcome evaluation the AUDIT questions were adapted to cover a period of three months as has been done by other researchers (Kunz et al., 2004). The 9-item PHQ-9 (Patient Health Questionnaire-9) was used to screen for probable depression (Patel et al., 2008).

Patients were invited to take part in the CAP trial if they were screened to be either harmful or dependent drinkers (AUDIT score ≥12 (Saunders et al., 1993)) – regardless of their score on the PHQ-9). Written or verbal informed consent was obtained from all participants; verbal consent was witnessed and formally recorded. In the context of this paper, data of all participants who agreed to take part in the CAP trial are analysed. Among those, participants were classified as comorbid if they exhibited at least mild symptoms of depression (PHQ-9 ≥5) in addition to their harmful or dependent drinking; otherwise, they were not.

A randomisation list stratified by PHC, created by an independent statistician, served as basis for random allocation of patients to either CAP plus Enhanced Usual Care (EUC) or EUC only. Using sequentially numbered opaque sealed envelopes, participants were randomized to one of the trial arms individually after baseline assessments had been completed. The screening, enrolment, consenting, baseline assessments and assignment of participants to trial arms based on the allocation sequence was done by trained and supervised field workers.

Participants were enrolled in the trial until the sample size specified in the PREMIUM study protocol (Patel et al., 2014) was reached. This sample size was powered to test the effectiveness of the CAP intervention in probable harmful drinkers (AUDIT score of 12–19) as described in an earlier publication (Nadkarni et al., 2017). The trial was not powered to estimate the effectiveness of the intervention for comorbid patients.

Screening started on Oct 28, 2013 and was completed on July 29, 2015. Outcome assessment took place both at 3- and 12-months post-randomisation with the last assessment taking place on Aug 30, 2016. The outcome assessors and lead investigators were blinded to the allocation status.

### 2.4 Intervention

Enhanced usual care (EUC) included provision of the WHO Mental Health GAP Action Programme (mhGAP) guidelines for management of AUD to the physician which also included recommendations on management of alcohol dependence in primary care and referral to specialist care as needed. In addition, the AUDIT screening results were provided to individual patients. Participants allocated to CAP received both CAP and EUC. CAP is a manualised psychological treatment informed by motivational interviewing and is delivered over a maximum of four sessions, though the optimal number is two. Sessions took place at weekly to fortnightly intervals and lasted 30−45 min. CAP consists of three phases: detailed assessment followed by personalised feedback in the initial phase; cognitive and behavioural skills and techniques, consisting of drink refusal skills, handling of peer pressure, problem-solving skills, and handling of difficult emotions in the middle phase; and learning how to manage potential or actual relapses in the ending phase. Additionally, those with alcohol dependence were given detailed information about and offered referral to local de-addiction centres where they could access services for medically assisted detoxification.

The CAP intervention was provided by 11 trained lay health workers (LHWs) with no previous background in mental health. All LHWs had at least completed high school education and were fluent in the vernacular languages used by study participants. Throughout the course of the trial, they underwent weekly supervision to ensure optimal delivery of the CAP treatment. A more detailed report of counsellor selection as well as training and supervision can be found in previous publications (Singla et al., 2014). A detailed description of the intervention and training material can be viewed online (https://nextgenu.org/course/view.php?id=229 and https://www.sangath.in/wp-content/uploads/2018/03/Counselling-for-Alcohol-Problems_Manual.pdf).

### 2.5 Measures

#### 2.5.1 Baseline measures

At baseline, basic sociodemographic parameters along with AUDIT and PHQ-9 scores were assessed. Additionally, readiness to change was measured using a five-point Likert scale (ranging from 1=” not useful at all” to 5=”very useful” and 1=”not at all” to 5=”already trying to change”, respectively).

#### 2.5.2 Acceptability and feasibility indicators

Over the course of the intervention, data were collected on acceptability and safety. These included the number of completed counselling sessions, session duration, homework completion, involvement of Significant Other (SO) in sessions and Serious Adverse Events (SAEs). A SAE was defined as either death due to any cause, unplanned hospitalization or suicidal behaviour. A patient was classified as a planned discharge if any of treatment completion was decided in consensus with the counsellor, drinking goals were achieved, or all four counselling sessions were completed.

#### 2.5.3 Clinical outcomes

A full overview of outcomes assessed in the PREMIUM trial can be found in the study protocol (Patel et al., 2014). The following outcomes were assessed 3 and 12 months after randomisation: Remission from depression (defined as PHQ-9 score < 5), AUD remission (defined as AUDIT score < 8) and abstinence. Abstinence was determined through the Time Line Follow Back (TLFB) tool, which has been shown to reduce bias in retrospectively assessing alcohol consumption over a specified timeframe (Sobell and Sobell, 1992). Abstinence was defined as not having consumed any alcohol over the 14 days prior to assessment.

### 2.6 Statistical analysis

We calculated frequencies and proportions for categorical variables and means and standard deviations for continuous variables at baseline.

For the analysis of process indicators, we employed descriptive statistics to present treatment acceptability and feasibility measures for comorbid and non-comorbid trial participants respectively. To test for differences between these two groups with regard to the process indicators, we conducted multivariable regression analyses adjusting for PHCs as a fixed effect.

Analysis of outcome data was conducted on an intention-to-treat basis with multiple imputation for missing outcome data using classification and regression trees. This was done because we assumed missing data to be missing not completely at random. We then used multivariable logistic regression to calculate adjusted Odds Ratios (aORs). In all regression models, we adjusted for PHCs as a fixed effect to allow for between-clinic clustering. For regression analyses using data of comorbid participants only, we additionally adjusted for education and marital status, which appeared imbalanced between the trial arms at baseline. We used likelihood ratio tests to assess statistical significance. All analysis was done using RStudio version 3.6.0.

## 3 Results

### 3.1 Recruitment and screening process Figure 1

**Fig. 1:**
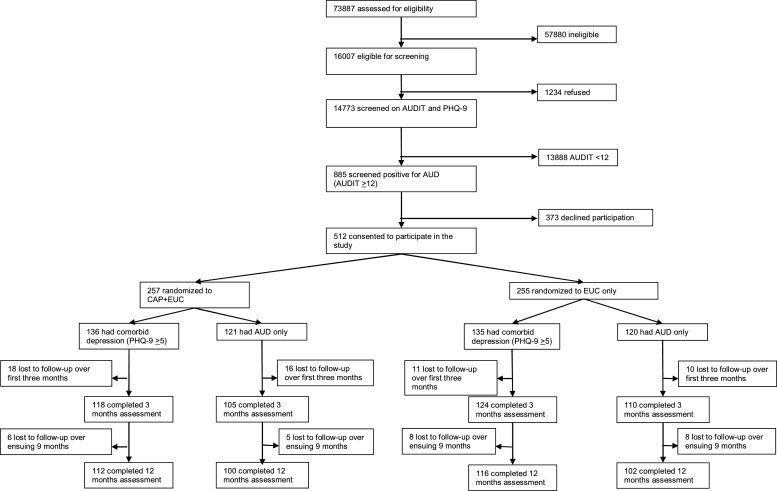
Flowchart of the Counselling for Alcohol Problems trial.

For the original PREMIUM trial, 73,887 patients were assessed for eligibility at ten PHCs, of whom 14,773 (20.0 %) were eligible and were screened for AUD and depression. Of these, 885 (6.0 %) participants screened positive for AUD (AUD≥12) overall, and 421 (2.8 %) also had at least mild symptoms of comorbid depression. 512 patients (57.9 %) agreed to take part in the CAP trial, with 257 (50.2 %) randomized to CAP + EUC and 255 (49.8 %) randomized to EUC only. Of the 512 CAP trial participants, 271 (52.9 %) were classified as comorbid with depression. Of these, 136 (50.2 %) were in the CAP + EUC trial arm, and 135 (49.8 %) were in the EUC-only arm. Of the 231 trial participants with AUD only, 121 (50.2 %) were in the CAP + EUC arm and 120 (49.8 %) were in the EUC-only arm.

### 3.2 Baseline measures

A summary of baseline measures for participants enrolled in the CAP trial is presented in Table 1.

**Table 1:** Baseline characteristics of comorbid participants and participants with AUD only.

Measure	Comorbid (n = 271)		AUD only (n = 241)	
	CAP + EUC (n = 136)	EUC only (n = 135)	CAP + EUC (n = 121)	EUC only (n = 120)
*Mean Age in years (SD)*	41.84 (11.8)	40.54 (10.2)	43.31 (11.5)	41.97 (11.5)
*Marital status (n, %)*				
Married	101 (74.3)	112 (83.0)	97 (80.2)	96 (80.0)
*Occupation (n, %)*				
Unemployed	23 (16.9)	20 (14.8)	14 (11.6)	18 (15.0)
*Education (n, %)*				
Completed secondary education or higher	41 (30.1)	30 (22.2)	34 (28.1)	38 (31.7)
*Patient’s expectation of usefulness of counselling (n, %)*				
A little/somewhat useful	23 (16.9)	29 (21.5)	24 (19.8)	25 (20.8)
Moderately useful to very useful	113 (83.1)	106 (78.5)	97 (80.2)	95 (79.2)
*Mean AUDIT score (SD)*	18.60 (5.3)	18.89 (5.4)	15.61 (3.6)	15.96 (4.2)
*Mean PHQ-9 score (SD)*	9.92 (4.5)	10.42 (4.4)	1.82 (1.4)	1.77 (1.44)
*Readiness to change (n, %)*				
Not at all/ little/ somewhat trying	22 (15.6)	13 (10.3)	23 (18.3)	24 (20.7)
Ready to already trying	114 (84.4)	122 (89.7)	98 (81.7)	96 (79.3)

*Notes.* AUDIT = Alcohol Use Disorder Identification Test, PHQ-9 = Patient Health Questionnaire-9.

Within the group of comorbid patients, those randomized to CAP + EUC were less likely to be married and more likely to have completed at least secondary education, while other parameters were found to be comparable between the two groups. Within the group of participants with AUD only, all measured parameters were evenly distributed (Table 1). Comparing all comorbid patients to the group of all patients with AUD only, mean AUDIT score was higher in the comorbid group compared to those with only AUD (18.75, SD = 5.3 versus 15.78, SD = 3.9, p < 0.001), and a greater readiness to change was observed (87.1 % of comorbid patients ready or already trying to change versus 80.4 % of non-comorbid patients, p = 0.06).

### 3.3 Acceptability, feasibility and safety

Within the group of comorbid patients, 128 (94.1 %) completed the first session of CAP, 96 (70.6 %) completed the second session, 65 (47.8 %) the third session and 28 (20.6 %) the fourth session, respectively. These proportions are comparable to those in the group with AUD only (93.4 %; 72.7 %; 46.3 %; 17.4 % respectively).

Key indicators describing the acceptability and feasibility of the CAP treatment for both comorbid and non-comorbid patients are displayed in the following (Table 2).

**Table 2:** Comparison between acceptability and feasibility indicators between comorbid patients and patients with AUD only.

Measure	Comorbid (n = 136)	AUD only (n = 121)	Effect measure (95 % CI)	p
Mean number of sessions (SD)	2.36 (1.2)	2.32 (1.1)	0.05^c^ (−0.24 – 0.34)	0.72
Mean duration of sessions in minutes (SD)	44.48 (9.5)	41.97 (9.6)	1.77^c^ (-0.64 – 4.18)	0.15
Homework completion^a^ (%)	73 (76.5)^a^	68 (80.0)^a^	0.88^d^ (0.40–1.92)	0.75
Planned discharge (n,%)	86 (63.2)	85 (70.2)	0.72^d^ (0.41–1.24)	0.24
SO involvement^b^ (n,%)				
Session 2	17 (18.5)^b^	13 (15.3)^b^	1.78^d^ (0.75–4.41)	0.20
Session 3	8 (12.5)^b^	4 (7.7)^b^	2.09^d^ (0.57–9.14)	0.29
Session 4	0 (0)	0 (0)	NA	NA

Notes: SO = Significant Other, a = among those assigned homework, b = among those attending the respective session, c = adjusted mean difference, d = adjusted odds ratio. All regression analyses controlling for PHC as fixed effect.

Comparing values for the comorbid cohort to those obtained for the group with AUD only suggests no evidence of differential acceptability and feasibility (Table 2).

Analysis of safety indicators showed that in the group of comorbid patients, the number of patients with at least one SAE was higher (n = 41, 15.1 % vs. n = 23, 9.5 %, aOR = 3.66, 95 % CI 1.38–11.56, p = 0.01). Among comorbid patients, those who received CAP + EUC treatment had a similar number of patients with at least one SAE as the group which received only EUC (n = 22, 16.2 % vs. n = 19, 14.0 %, aOR = 0.84, 95 % CI 0.43–1.64, p = 0.62).

### 3.4 Preliminary analysis of effectiveness

Follow-up rates were high at both 3 months and 12 months. Of the comorbid patients, 242 (89.3 %) completed a 3 month outcome assessment and 228 (84.8 %) completed the 12 month assessment. The proportions were similar in the AUD-only participants (89.2 % and 84.8 % respectively). Of the baseline measures, only age proved a significant predictor of drop-out: patients below 40 years were less likely to drop out after 3 months and 12 months respectively (OR = 0.41, 95 % CI 0.23–0.73; OR = 0.53, 95 % CI 0.32–0.85).

Among the comorbid participants, there was no evidence of a difference between the two arms for the two AUD-related outcomes at any time point, though the proportion of participants with AUD remission and of non-drinkers was consistently higher among those who received CAP + EUC vs EUC-only (Table 3). There was no evidence of an intervention effect on remission from depression.

**Table 3:** Outcome data for comorbid and non-comorbid CAP participants per trial arm at 3 and 12 months follow-up.

	Comorbid				AUD only			
Outcome at 3 months follow-up	CAP + EUC (n = 118)	EUC (n = 124)	aOR (95 % CI)	p	CAP + EUC (n = 105)	EUC (n = 110)	aOR (95 % CI)	p
AUD remission (AUDIT < 8) (n,%)	37 (31.4)	29 (23.4)	1.51 (0.84–2.74)	0.17	38 (36.2)	24 (21.8)	1.84 (1.00–3.41)	0.05
Non-drinker (n,%)	47 (39.8)	38 (30.6)	1.48 (0.86–2.55)	0.16	42 (40.0)	12 (10.9)	4.64 (2.32–9.28)	<0.01
PHQ-9 remission (PHQ-9 < 5) (n,%)	51 (43.2)	61 (49.2)	0.74 (0.43–1.27)	0.28	NA	NA	NA	NA

Outcome at 12 months follow-up	CAP + EUC (n = 112)	EUC (n = 115)	aOR (95 % CI)	p	CAP + EUC (n = 99)	EUC (n = 102)	aOR (95 % CI)	P
AUD remission (AUDIT < 8) (n,%)	48 (42.9)	30 (26.1)	1.69 (0.96–3.01)	0.08	53 (53.5)	32 (31.4)	2.47 (1.34–4.57)	<0.01
Non-drinker (n,%)	47 (42.0)	35 (30.4)	1.39 (0.76–2.50)	0.28	42 (42.4)	24 (23.5)	2.17 (1.18–3.98)	0.01
PHQ-9 remission (PHQ-9 < 5) (n,%)	62 (55.4)	56 (48.7)	1.08 (0.62–1.87)	0.79	NA	NA	NA	NA

*Notes*. CAP = Counselling for Alcohol Problems, EUC = Enhanced Usual Care. AUDIT = Alcohol Use Disorder Identification Test, PHQ-9 = Patient Health Questionnaire-9, aOR = adjusted Odds Ratio. All aORs adjusted for Primary Healthcare Centre; aORs in comorbid group additionally adjusted for marital status and education. Missing outcome values for all variables were imputed via multiple imputation using classification and regression trees.

Among patients with AUD only, there was strong evidence of an intervention effect on AUD remission and non-drinking at both 3 months and 12 months (Table 3). Additionally, there was strong evidence suggesting that the effect of CAP on abstinence at 3 months follow-up was modified by the presence of comorbidity (aOR of interaction term 3.23, 95 % CI 1.34–7.79, p < 0.01), whereas for all other outcomes, this was not the case.

## 4 Discussion

Our results suggest that identifying patients with comorbid AUD and depression in a primary care setting is feasible, and that the acceptability and safety of the CAP treatment among these patients is generally satisfactory and comparable to a group of patients with AUD without symptoms of comorbid depression. Exploratory findings from the group of comorbid patients suggest that although delivering CAP does not seem to result in more frequent remission from depression compared to EUC only, it may have a positive effect on AUD-related outcomes. Among participants with AUD only, there was strong evidence for a positive effect of CAP on both remission from AUD and abstinence.

This study makes an important contribution to the existing literature since, to the best of our knowledge, it is the first to test such a treatment for patients with comorbid depression and AUD in a LMIC-setting. Our findings add to the evidence supporting the feasibility of a task-sharing approach in countries where insufficient resources hamper the provision of essential treatment for mental disorders (Chibanda et al., 2015; Nadkarni et al., 2017; Patel et al., 2017). In this study, even in the case of comorbid patients with potentially complex clinical pictures, NSHWs were able to deliver a brief treatment safely while overall maintaining satisfying engagement comparable to non-comorbid participants in the trial. In particular, drop-out was low in this study, which may be partly explained by direct recruitment of participants at PHCs and the delivery of CAP by NSHWs in vernacular languages.

Among the comorbid patients, the proportion classified as abstinent or remitted from AUD was found to be higher in the group of patients who received CAP + EUC compared to only EUC at both 3 months and 12 months follow-up. This trial was not powered to demonstrate the effectiveness of CAP in a comorbid sample, but provides an indication of a positive effect of CAP + EUC in comorbid patients to be investigated in future studies. The effectiveness of CAP appears higher among patients with AUD only, for both AUD-related outcomes, and at both 3 and 12 months follow-up; however, strong evidence for a modification of the effect of CAP by the presence of comorbidity was only found for abstinence at 3 months follow-up.

Interestingly, this effect measure modification is mostly explained by a differential response to EUC only rather than to CAP + EUC. While among participants who received CAP + EUC, a similar proportion of patients was classified as abstinent in the comorbid and non-comorbid group at 3 months follow-up, a lower proportion of patients with AUD only (10.9 %) reported abstinence compared to comorbid patients (30.9 %) among those who received EUC only. One possible explanation for this observation is that comorbid patients were more aware that they had a health problem than patients with AUD only, resulting in a greater readiness to change. This has also been found in a previous investigation in a general practice high-income setting (Grothues et al., 2005) and is partly supported by the data, which indicate that more comorbid patients were “already trying to change” their drinking habits at baseline compared to non-comorbid patients. This may have made them more motivated to accomplish abstinence prior to assessment, while other potentially harmful drinking patterns (as measured via the AUDIT) may nonetheless have remained partly unchanged.

We also conducted an additional exploratory analysis to investigate whether the effect of CAP in the group of comorbid patients is modified by the severity of depression, with results indicating that CAP may be effective in patients with mild comorbid depression, but may have no effect in patients with more severe depression (see appendix). This requires further testing in larger studies as well as an improved qualitative understanding as to why this may be the case, ultimately informing guidelines on which disorder should be treated first in patients with comorbid AUD and depression. Interestingly, the stratified analysis also reveals a pattern similar to the one mentioned above: patients with less severe (i.e. mild) symptoms of depression seem to have responded consistently worse to EUC than those with at least moderate depression. With no difference in baseline intention to change between the two groups, this is a curious observation. Given that more severe symptoms of depression likely reflect dysfunctional thought patterns reducing a patient’s ability to improve their consumption habits (Hasin et al., 2002), one might expect more severe symptoms of depression to be associated with a worse response to EUC only. This observation may well be entirely due to chance; however, it may be worth investigating further in future investigations.

Looking at depression as the second outcome, study data suggest that the CAP intervention did not significantly improve depressive symptoms in comorbid participants compared to those who received EUC only. This finding is – once again – subject to the small sample size, but casts some doubt on the question of whether a treatment specifically tailored to addressing AUD may also be effective in the treatment of depression. Future research should consider developing and testing interventions which cater specifically to the needs of comorbid patients, thus addressing both disorders similarly and accounting for their possible interrelation.

Data from this RCT also give insight into the prevalence of comorbid AUD and depression in a primary care setting – a question mostly unexplored in India. The overall prevalence of comorbid AUD and depression was 2.8 % in this study population – however, this is sensitive to the PHQ-9 cut-off used to identify comorbid depression. In case of a higher cut-off (PHQ-9 ≥15) to identify at least moderately severe depression, this number goes down to 0.4 % - an estimate similar to community-level data from the NMHS (Gururaj et al., 2016), which used a different assessment tool (the Mini International Neuropsychiatric Interview). From a practical standpoint, this makes recruitment of comorbid patients in the given setting challenging. Researchers working in this field should explore different settings such as de-addiction centres, where a higher prevalence of comorbidity can be expected (Vohra et al., 2003). Furthermore, baseline data of this trial support the hypothesis of a co-occurrence of AUD and depression: Among those who screened positive for AUD, 47.6 % also screened positive for at least mild comorbid depression and 7.1 % for at least moderately severe depression. Compared to prevalence data for at least moderately severe depression in the same setting (2.7 % overall (Patel et al., 2017)) and India-wide in the NMHS (2.37 % in men (Gururaj et al., 2016)), this is substantially higher and can be considered further evidence that the two disorders co-occur also in a LMIC-setting. As already noted, we also find that suffering from both AUD and depression is associated with significantly higher AUDIT scores, which should be considered further evidence that the experience of comorbidity is qualitatively different from just the sum of two disorders.

This study has several important limitations: First, it was not powered to detect an intervention effect. Second, findings from this trial apply to men only, as women were not screened for AUD in the PREMIUM trial. However, given the very low prevalence of AUD in Indian women (Gururaj et al., 2016), it seems unlikely that many have comorbid AUD and depression. Third, we relied on self-reported data for measuring alcohol consumption. Even though the AUDIT tool has been validated in the India context (Pal et al., 2004), we cannot rule out that alcohol intake has been reported differentially depending on treatment group. Fourth, we did not assess the presence of further comorbidities such as anxiety. If comorbid patients are more likely to suffer from e.g. anxiety, this may have impacted their response to CAP. Finally, we did not assess whether participants received additional treatment for either AUD or depression. However, given that most cases of depression and AUD remain unidentified and untreated in primary care in India (Gururaj et al., 2016), this is unlikely to have had a strong effect.

## 5 Conclusions

In conclusion, this study may be considered an encouraging starting point for systematically developing NSHW-delivered psychological treatments for the underserved and vulnerable population of comorbid patients with AUD and depression in India. Even though data from this study only allow for tentative claims about and partly cast doubt on the effectiveness of the AUD-only focused treatment tested here, they show that it can be safely delivered in a population of comorbid patients in an LMIC primary care setting. Hence, with accumulating knowledge through larger trials in settings with higher prevalence of comorbidity and more specifically tailored interventions to meet the needs of comorbid patients, brief psychological treatments delivered by NSHWs could become an important cornerstone in addressing the treatment gap for patients with comorbid depression and AUD in India.

## Author declaration

Authors wish to confirm that there are no known conflicts of interest associated with this publication and there has been no significant financial support for this work that could have influenced its outcome.

Authors confirm that the manuscript has been read and approved by all named authors and that here are no other persons who satisfied the criteria for authorship but are not listed.

Authors further confirm that the order of authors listed in the manuscript has been approved by all of them.

Authors confirm that they have given due consideration to the protection of intellectual property associated with this work and that there are no impediments to publication, including the timing of publication, with respect to intellectual property. In so doing they confirm that they have followed the regulations of their institutions concerning intellectual property.

Authors understand that the Corresponding Author is the sole contact for the Editorial process (including Editorial Manager and direct communications with the office). He is responsible for communicating with the other authors about progress, submissions of revisions and final approval of proofs. Authors confirm that they have provided a current, correct email address which is accessible by the Corresponding Author.

## Contributors

JS and AN drafted the report; JS conducted the statistical analysis. HAW provided feedback on the manuscript and the statistical analysis. RV and VP provided feedback on the manuscript. AN, HAW, RV and VP contributed to the design, implementation and evaluation of the original PREMIUM trial. All authors have approved the final article.

## Role of funding source

The CAP trial was funded by a Wellcome Trust Senior Research Fellowship grant to Vikram Patel (091834).

## Data statement

The data that support the findings of this study are available from the corresponding author, AN, upon reasonable request.

## Declaration of Competing Interest

The authors report no declarations of interest.
