# Supplementary material for: A lay-counsellor delivered brief psychological treatment for men with comorbid Alcohol Use Disorder and depression in primary care: Secondary analysis of data from a randomized controlled trial
Source: Drug Alcohol Depend. 2021 Oct 1;227:108961. doi: 10.1016/j.drugalcdep.2021.108961 (PMC8504199; doi:10.1016/j.drugalcdep.2021.108961)
Supplement: Supplementary file 1 [file mmc1.docx]

**Appendix**

| Table 4. Outcome data of CAP participants with mild comorbid depression and with moderate and severe comorbid depression per trial arm at 3 and 12 months follow-up. | | | | | | | | | |
| --- | --- | --- | --- | --- | --- | --- | --- | --- | --- |
|  | Mild depression | | | |  | Moderate & severe depression | | | |
| Outcome at 3 months  follow-up | CAP + EUC  (n=66) | EUC  (n=56) | aOR (95% CI) | p |  | CAP + EUC (n=52) | EUC (n=68) | aOR (95% CI) | p |
| AUD remission  (AUDIT < 8) (n,%) | 23 (34.8) | 8 (14.3) | 3.03 (1.11 – 8.28) | 0.03 |  | 14 (26.9) | 21 (30.9) | 0.87 (0.36 - 2.08) | 0.76 |
| Non-drinker (n,%) | 28 (42.4) | 11 (19.6) | 3.04 (1.21 – 7.65) | 0.02 |  | 19 (36.5) | 27 (39.7) | 0.86 (0.39 – 1.88) | 0.7 |
| PHQ-9 remission  (PHQ-9 < 5) (n,%) | 34 (51.5) | 34 (60.7) | 0.68 (0.31 – 1.46) | 0.32 |  | 17 (32.7) | 27 (39.7) | 0.61 (0.26 - 1.42) | 0.25 |
| Outcome at 12 months  follow-up | CAP + EUC (n=61) | EUC (n=54) | aOR (95% CI) | p |  | CAP + EUC  (n=51) | EUC  (n=61) | aOR (95% CI) | P |
| AUD remission  (AUDIT < 8) (n,%) | 32 (52.5) | 12 (22.2) | 2.75 (1.10 – 6.87) | 0.03 |  | 16 (31.4) | 18 (29.5) | 1.01 (0.42 – 2.45) | 1 |
| Non-drinker (n,%) | 28 (45.9) | 13 (24.1) | 1.87 (0.73 – 4.76) | 0.19 |  | 19 (37.3) | 22 (36.1) | 0.85 (0.36 – 2.01) | 0.71 |
| PHQ-9 remission  (PHQ-9 < 5) (n,%) | 40 (65.6) | 33 (61.1) | 0.97 (0.42 – 2.22) | 0.93 |  | 22 (43.1) | 23 (37.7) | 0.95 (0.43 - 2.14) | 0.91 |
| *Notes*. CAP= Counselling for Alcohol Problems, EUC= Enhanced Usual Care. AUDIT= Alcohol Use Disorder Identification Test, PHQ-9= Patient Health Questionnaire-9, aOR = adjusted Odds Ratio. All aORs adjusted for Primary Healthcare Centre, marital status and education. Missing outcome values for all variables were imputed via multiple imputation using classification and regression trees. | | | | | | | | | |
